# A novel hybrid model for six main pollutant concentrations forecasting based on improved LSTM neural networks

**DOI:** 10.1038/s41598-022-17754-3

**Published:** 2022-08-24

**Authors:** Shenyi Xu, Wei Li, Yuhan Zhu, Aiting Xu

**Affiliations:** 1 School of Statistics and Mathematics, Zhejiang Gongshang University, No.18 Xuezheng Street, Xiasha Higher Education Park, Hangzhou, Zhejiang China; 2 Present Address: Collaborative Innovation Center of Statistical Data Engineering, Technology & Application, Zhejiang Gongshang University, Hangzhou, China

**Keywords:** Environmental social sciences, Environmental monitoring, Environmental sciences, Environmental impact

## Abstract

In recent years, air pollution has become a factor that cannot be ignored, affecting human lives and health. The distribution of high-density populations and high-intensity development and construction have accentuated the problem of air pollution in China. To accelerate air pollution control and effectively improve environmental air quality, the target of our research was cities with serious air pollution problems to establish a model for air pollution prediction. We used the daily monitoring data of air pollution from January 2016 to December 2020 for the respective cities. We used the long short term memory networks (LSTM) algorithm model to solve the problem of gradient explosion in recurrent neural networks, then used the particle swarm optimization algorithm to determine the parameters of the CNN-LSTM model, and finally introduced the complete ensemble empirical mode decomposition of adaptive noise (CEEMDAN) decomposition to decompose air pollution and improve the accuracy of model prediction. The experimental results show that compared with a single LSTM model, the CEEMDAN-CNN-LSTM model has higher accuracy and lower prediction errors. The CEEMDAN-CNN-LSTM model enables a more precise prediction of air pollution, and may thus be useful for sustainable management and the control of air pollution.

## Introduction

Air pollution can significantly affect air quality^1^, More than 90% of the world’s population resides in places where air pollution levels surpass the limits specified by the World Health Organization (WHO)^2^. As the largest developing country, China has been suffering from serious air pollution for years in response to the rapid industrialization and urbanization^3^, which has led to a dramatic increase in the emissions of both ambient air pollutants and greenhouse gases^4^. The major air pollutants in China are PM2.5 (particles ≤ 2.5 μm in aerodynamic diameter), PM10 (particles ≤ 10 μm in aerodynamic diameter), sulfur dioxide (SO2), nitrogen oxide (NO2), carbon monoxide (CO), and ozone (O3)^5^.Almost every major Chinese city exceeds the limits for air pollutants recommended by the WHO, leading to approximately 1.1–1.6 million premature deaths annually^6^. Air pollution can also lead to huge direct and indirect losses to the social economy. In severe haze pollution events, public and private transportation can be severely affected by a reduction in visibility. Therefore, measuring, monitoring, and predicting air quality are vital for achieving the eventual reduction of haze risks in practical life^7^.

Many cities in China have experienced serious local pollution events owing to coal mining, urbanization, excessive coal consumption, and the development of heavy industries (such as iron, steel, and cement)^8^. In particular, according to statistics, the bottom 20 cities of the 168 key cities in China, ranked in terms of air quality in 2020, are facing serious air pollution problems^9^. To facilitate the management and research of China's regional compound air pollution, we targeted these cities for the accurate and real-time prediction of air pollutants.

With the maturity of various machine learning methods, deep learning models based on neural networks have been used in air pollution research. Deep learning methods based on long short-term memory (LSTM) artificial neural networks, radial basis functions (RBFs), back propagation (BP) neural networks, and support vector machines (SVMs) have been used by many scholars to study the non-linear relationship between air quality and meteorological data^10–12^. These methods are divided into two main types: single machine learning and hybrid machine learning models. Table 1 summarizes studies of air pollutants and air pollution forecasting models published in the past three years. Hybrid machine learning models have received considerable attention, as presented in Table 1.

**Table 1:** Studies on forecasting air pollutants using different models.

Study	Research subject	Type	Method
Feng^13^	SO2, NO2, CO, PM2.5, PM10 and O3	S	RNN;RF
Li et al.^14^	PM2.5 and NOx	S	RF; BRT; SVM; XGBoost; GAM
Yan et al*.*^15^	PM2.5	S	CNN;LSTM
Awan et al.^16^	CO, NO, NO2, NOx, and O3	S	LSTM
Dairi et al.^17^	NO2, O3, SO2, and CO	H	IMDA-VAE
Lu et al.^18^	PM2.5	H	OR-ELM-AR
Du et al.^19^	PM2.5	H	1D-CNNs; Bi-LSTM
Yafouz et al.^20^	PM2.5, O3, SO2, NO2, CO, AQI	H	SVR-LSTM

*S*: Single machine learning method, *H*: Hybrid machine learning model.

Therefore, the principal objective of this study was to develop a new and highly accurate air pollution forecasting method. In this study, we developed a new hybrid model based on complete ensemble empirical mode decomposition of adaptive noise (CEEMDAN), convolutional neural network (CNN), and LSTM neural networks for air pollution forecasting.

First, we found that the LSTM model has shown potential in adapting to different types and representations of data, recognizing sequential patterns over a long time span, and capturing complex nonlinear relationships^21^. Thus, it has been applied to various time-series prediction fields, including stock price movement^22^, ocean wave height series^23^, and air pollutant prediction^24–26^. However, the commonly used forecasting methods at present often use a single forecasting model to model the time-series; as such, they cannot intuitively reflect the nonlinearity of the corresponding series. Therefore, the accuracy of the corresponding prediction results is lacking. The combined forecasting model effectively solves the above problems, and the accuracy of the overall forecasting results can be achieved be completely considering to the advantages of each forecasting model^27^. Therefore, when the time-series data have the characteristics of significant randomness and rich characteristic information, the concept of individual response should be adopted to explore the prediction method based on the adaptive noise-added ensemble empirical mode decomposition (CEEMDAN). The air pollution time series problem is transformed into a number of component prediction problems with significant regularity, and the component prediction results are then merged and analyzed to obtain a higher-precision prediction value, thereby making the modeling easier and more accurate^28^.

In accordance with the above ideas, this study proposes a deep learning model based on LSTM of CEEMDAN and CNN for evaluating the meteorological and air pollution data of major cities with serious air pollution problems in China. Moreover, the particle swarm optimization (PSO) algorithm is used to determine the parameters of the CNN-LSTM model. To study and predict the air pollution value, we aimed to find the optimal deep learning model suitable for this type of data. Simultaneously, we concentrate on estimating the health burden associated with air pollution. We comprehensively investigated the health burden attributable to the long-term exposure to PM2.5, PM10, SO2, NO2, CO, and O3 in China by 2021. We also aimed to estimate the premature deaths attributable to the long-term exposure to the above ambient air pollutants in Chinese cities. The results will therefore provide a scientific basis to formulate relevant policies for air pollution control projects. It also provides a reference for areas around the world with increasingly prominent air pollution problems in basins owing to the high-density population layout and high-intensity development and construction.

## Materials and methods

The main methodology of this study involved deep learning and frequency decomposition algorithms. Moreover, to understand the proposed method, first, it is crucial to understand the constituting models, as well as how they learn or perform. The air pollution forecasting models in this study include CEEMDAN, CNN, LSTM, and PSO. A brief description of these methods is given below.

### Complete ensemble empirical mode decomposition with adaptive noise

The empirical mode decomposition (EMD) algorithm is a signal analysis method originally proposed by Huang et al.^29^. It is an adaptive data processing or mining method that is suitable for the processing of nonlinear and non-stationary time series. It is also essentially a smoothing process of data series or signals^30^. In EMD, any given complex signal can be empirically decomposed into a collection of basic oscillatory components, called intrinsic mode functions (IMFs). The IMF represents the oscillation mode of the original signal^31^. The original signal $x(t)$ can be reconstructed by the following formula:

1 $$x\left( t \right) = \mathop \sum \limits_{i = 1}^{n} c_{i} \left( t \right) + r_{n} \left( t \right)$$

where ${c}_{i}\left(t\right)$ is the $i$ th IMF (i.e. local oscillation) and ${r}_{n}\left(t\right)$ is the $i$ th residue (i.e. local trend).

The EMD method can ideally be applied to the decomposition of any type of time series (signal) because of its obvious advantages over previous smoothing methods in dealing with non-stationary and non-linear data^32,33^. To overcome the problem of mode mixing in EMD and solve the problem of IMF component alignment during ensemble averaging, Torres improved CEEMD from the decomposition process and added white noise, and then proposed a complete ensemble empirical mode decomposition of adaptive noise^34^. In the new CEEMD, white noise is added in pairs to the original data (i.e. one positive and one negative) to generate two sets of ensemble IMFs.

2 $$\left( {\begin{array}{*{20}c} {M_{1} } \\ {M_{2} } \\ \end{array} } \right) = \left[ {\begin{array}{*{20}c} 1 \\ 1 \\ \end{array} \begin{array}{*{20}c} 1 \\ { - 1} \\ \end{array} } \right]\left[ {\begin{array}{*{20}c} S \\ N \\ \end{array} } \right]$$

where $S$ is the original data data; $N$ is the added white noise; ${M}_{1}$ is the sum of the original data with positive noise, and ${M}_{2}$ is the sum of the original data with the negative noise. There is less residual noise in the inherent modal components, which effectively reduces the reconstruction error, and a global stopping standard exists at each stage of the decomposition. The decomposition efficiency in this method was the highest^35^.

This study uses the CEEMDAN algorithm to decompose non-stationary air pollution series data to form a series of IMF subsequences and residual terms (RES) with different frequency characteristics.

### Convolutional neural network

A CNN is a neural network used to process data with a known grid-like topology^36^. A CNN is a feed-forward neural network whose basic structure is determined by the input, convolutional, pooling, fully connected, and output layers^37^. The convolutional layer is the core of the CNN, where the convolutional kernel ${C}_{j}$ is used to extract the internal features.

3 $$C_{j} = \sigma \left( {\sum A_{i} \otimes \omega_{i} + b_{i} } \right)$$

where ${A}_{i}$ represents the input, $\otimes$ represents a convolution operator, $\sigma$ represents the activation function (where ReLU is selected), ${\upomega }_{i}$ is the weight of the kernel linked to the $i$ th feature map, and ${b}_{i}$ represents the bias matrix.

**Figure 1:**
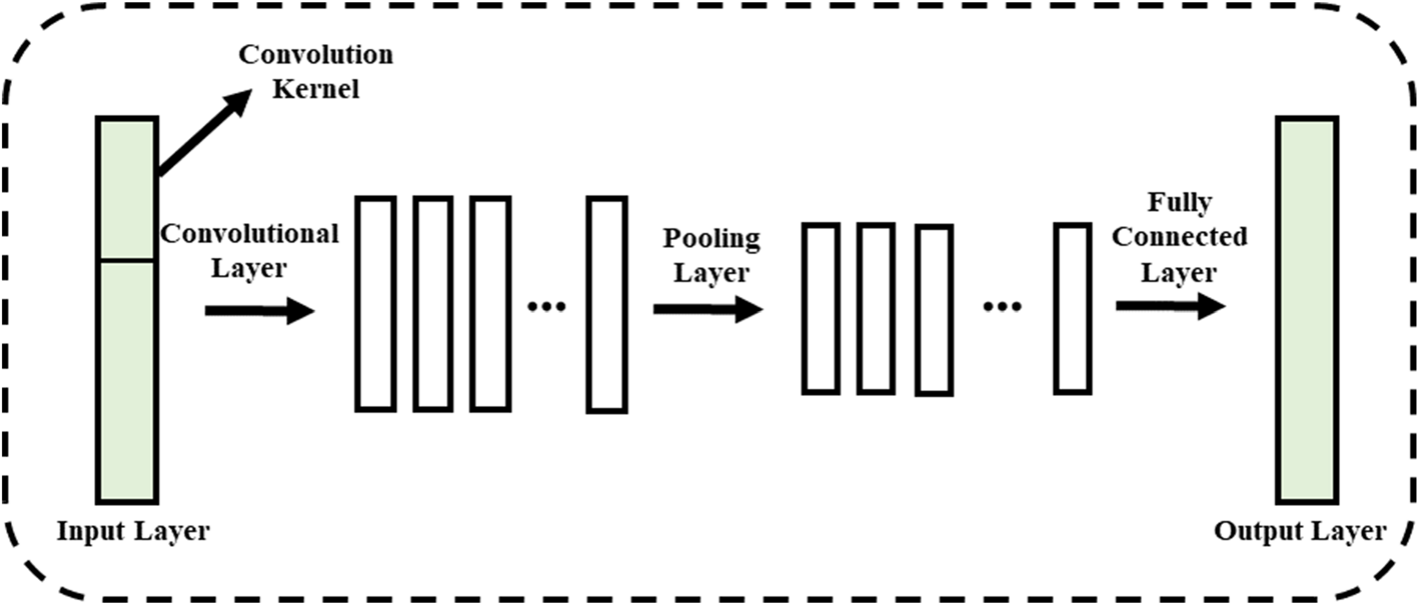
Structure of the one dimensional convolutional neural network.

The pooling layer is mainly used to pool the data after the sniper operation. Its main function is to compress the data, remove unnecessary information, effectively improve the generalization ability of the network, and increase the calculation speed^38,39^. Each node of the fully connected layer is connected to all nodes of the upper layer, which is used to integrate the comprehensive features extracted from the front and aid in the prediction of the subsequent LSTM layer^40^. The structure of a one-dimensional convolutional neural network is shown in Fig. 1.

### Long short-term memory model

Long and short-term memory (LSTM)^41^ neural networks are special recurrent neural networks that can learn dependent information for a long time and effectively avoid the phenomenon of a disappearing gradient^42^. It is a machine-learning architecture that allows the model to “learn” over many time steps. Additionally, it can root the memory cell in the neural nodes of the hidden layer of the cyclic neural network to record historical information; by adding three gate structures (input, forget, and output), the historical information can be realized^43^.

**Figure 2:**
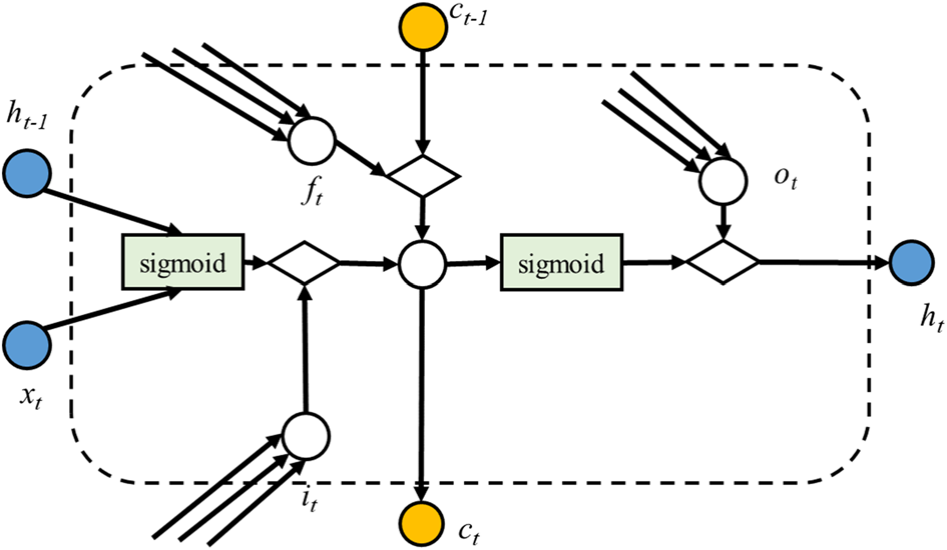
Structure of the long- and short-term memory neural network cell.

As shown in Fig. 2, when setting the input sequence to $x({x}_{1},{x}_{2},\dots ,{x}_{t})$, the state of the hidden layer is ${(h}_{1},{h}_{2},\dots ,{h}_{t})$, and the state update and output of the memory unit can be summarized as

4 $$i_{t} = sigmoid\left( {W_{hi} h_{t - 1} + W_{xi} x_{t} } \right)$$

5 $$f_{t} = sigmoid\left( {W_{hf} h_{t - 1} + W_{xf} x_{t} } \right)$$

6 $$c_{t} = f_{t} \odot c_{t - 1} + i_{c} \odot \tanh \left( {W_{xc} x_{t} + W_{hc} h_{t - 1} } \right)$$

7 $$o_{t} = sigmoid\left( {W_{xo} x_{t} + W_{ho} x_{t - 1} + W_{co} c_{t} } \right)$$

8 $$h_{t} = o_{t} \odot tanh\left( {c_{t} } \right)$$

where “$\odot$ ” denotes the Hadamard product; ${i}_{t}$, ${f}_{t}$, and ${o}_{t}$ are the output of different gates; ${c}_{t}$ is the vector for the cell state; ${h}_{t-1}$ is the output information of the hidden layer unit at the previous moment; ${h}_{t}$ is the new state of memory cell; ${W}_{h}$, ${W}_{x}$, and ${W}_{c}$ are the weights of the corresponding gate; and $sigmoid$ and $\mathrm{tanh}$ are the two different activation functions, respectively.

As air pollution data from ground monitoring sites are usually in a time series format, air pollution can be modeled by considering the time-dependent patterns ^44^. Feed-forward neural networks (FNNs) have been commonly used in previous studies to predict air pollution. However, these models cannot consider the time dependency of the parameters. Sequence modeling facilitates the excavation of temporal dynamic features in historical data and enusres better predictions^45^. Compared with the FNN, recurrent neural networks (RNN) are designed to deal with time-series data; however, this technique experiences vanishing or exploding gradient problems^46^, and LSTM can be used to overcome this problem. In this study, we chose LSTM for air pollution prediction as it extracts representative features from historical air pollution data and obtains further representations of the merged features to generate predictions.

### Particle swarm optimization

Particle swarm optimization (PSO) is an evolutionary computation technique developed by Kennedy and Eberhart in 1995^47^. This algorithm is a swarm intelligence optimization algorithm that simulates the foraging behavior of bird swarms and adjusts its own speed and position to optimize it until it meets the convergence termination condition^48,49^. All particles in the swarm stay in the set search space, as shown in Eqs. (6) and (7):

9 $$V_{i,j}^{t + 1} = \omega V_{i,j}^{t} - c_{1} r_{1,i,j}^{t} \left( {\hat{y}^{t} - x_{i,j}^{t} } \right) + c_{2} r_{2,i,j}^{t} \left( {y_{i,j}^{t} - x_{i,j}^{t} } \right)$$

10 $$x_{i,j}^{t + 1} = x_{i,j}^{t} + V_{i,j}^{t + 1}$$

where ${V}_{i,j}^{t}$ is the velocity of particle $i$ at generation $t$, and $j$ is the dimension; ${x}_{i,j}^{t}$ is the position of particle $i$; ${c}_{1}$ and ${c}_{2}$ are cognitive and social coefficients; ${y}_{i,j}^{t}$ is the best value in the group at generation $t$; ${\widehat{y}}^{t}$ is the best value of all of the best values from different groups; and ${r}_{1,i,j}^{t}$ and ${r}_{2,i,j}^{t}$ are uniformly distributed random numbers in the interval [0,1]. Furthermore, the concept of inertia weight $\omega$ is developed to obtain better control exploration and exploitation of the searched particles.

PSO has several advantages over other metaheuristic techniques in terms of its simplicity, convergence speed, and robustness. It converges to global or near-global optima, irrespective of the shape or discontinuities of the cost function. As PSO can prevent the network convergence from falling into the local best solution, it can be selected to optimize the LSTM input layer weights^50^. Most previous studies on PSO systems have provided empirical results and conducted informal analyses^47,51,52^. Many studies have shown that the PSO algorithm can improve the prediction accuracy by optimizing the LSTM model^53–55^. Thus, this study initially proposes an enhanced PSO-based LSTM model, which is used to forecast air pollution.

## Proposed air pollution forecasting model

### The CEEMDAN-CNN-LSTM model

Air pollution data are a time series characterized by complex instability, nonlinearity, and periodic uncertainty, which are affected by many factors. As mentioned above, LSTM has a strong modeling and analysis ability for processing time-series data. The performance of the LSTM model in time-series analysis is extraordinary^47^. However, the LSTM model only extracts the temporal features of the flames, whereas turbulent flames characterize both the temporal and spatial evolution. A CNN is a deep learning network wherein the local and overall features of the input data can be constantly extracted using nonlinear mapping^56,57^. It can extract the spatial features of the flames. Therefore, we proposed a combination of CNN and LSTM models. CNN-LSTM has been extensively employed for time-series forecasting. However, determining the structure is difficult, and often falls into a local minimum^58^. The CEEMDAN method can divide the singular values into separated IMFs and determine the general trend of the real time series; thus, it can help determine the characteristics of the complex non-linear or non-stationary time-series data^59^. This can effectively reduce unnecessary interactions among singular values and improve the performance when a single kernel function is used in forecasting^60^. This section proposes a model that combines the CEEMDAN and CNN-LSTM models for air pollution prediction.

**Figure 3:**
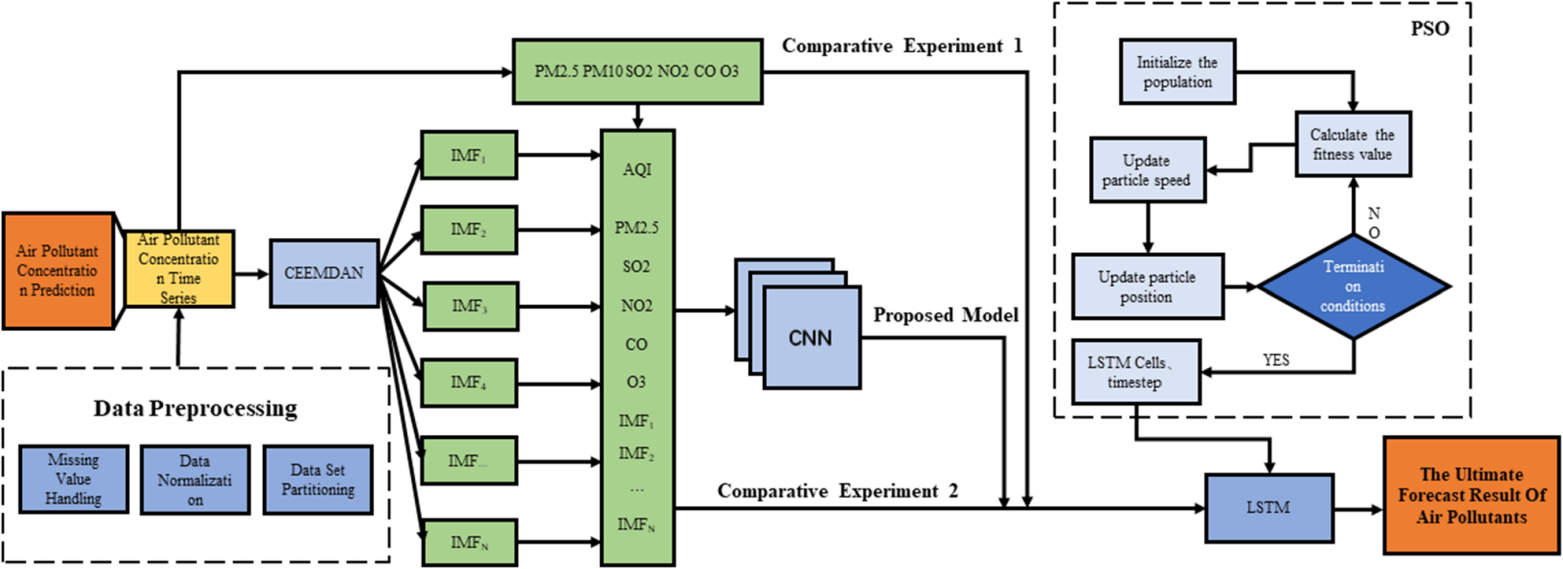
Flow chart of air pollution prediction based on the integrated CEEMDAN-CNN-LSTM model.

As shown in Fig. 3, in this study, the CEEMDAN algorithm was used to decompose the data of air pollution change, measured by the air quality monitoring station, to obtain a limited number of IMFs. Subsequently, we used the CNN-LSTM model to learn and predict the short-term time series of each IMF component, and added the predicted values of each IMF component to obtain the final prediction result.

Finally, PSO is used to optimize the hyperparameters of LSTM because of its simplicity and ease of implementation^61^. The core idea of the PSO algorithm is to first initialize a set of random solutions and then iteratively find the optimal solution^62^. The PSO algorithm can enable the LSTM model to accurately and quickly determine the optimal parameters according to the characteristics of the air pollution data, and realize an effective combination of the network structure of the LSTM model and the features of the air pollution data^63^.

### Model fitting and validation

To evaluate the predictive ability of the models, two indices, namely, the root mean square error (RMSE) and mean absolute error (MAE) were calculated in this study. In general, the smaller the RMSE, MAE, and ${R}^{2}$, the more accurate the model. RMSE, MAE, and ${R}^{2}$ are defined in Eqs. (11)–(13), respectively.

11 $$RMSE = \sqrt {\frac{{\mathop \sum \nolimits_{i = 1}^{n} (y_{i} - f_{i} )^{2} }}{n}}$$

12 $$MAE = \frac{{\sum\nolimits_{i = 1}^{n} {\left| {(f_{i} - y_{i} )} \right|} }}{n}$$

13 $$R^{2} = \frac{{\mathop \sum \nolimits_{i = 1}^{n} \left( {f_{i} - \overline{y}} \right)^{2} }}{{\mathop \sum \nolimits_{i = 1}^{n} \left( {y_{i} - \overline{y}} \right)^{2} }}$$

where $n$ is the number of data points, ${y}_{i}$ is the measured aqueous air pollution, $\overline{y }$ is the average of the real values, and ${f}_{i}$ is the air pollution simulated by the model.

## Case study

### Study area and data set

According to historical research, air pollution is highly correlated with six air pollutants (PM2.5, PM10, NO2, CO, O3, and SO2)^15,64–66^. Therefore, this study investigated 20 cities with the worst air quality in China and selected the most representative 6 cities according to their primary pollutants, economic conditions, and geographical factors to prove the validity and robustness of the hybrid model. The final choices were Xinxiang (main air pollutant: PM2.5), Taiyuan (PM10), Zibo (SO2), Handan (NO2), Binzhou (O3), and Jinan (CO). In this study, data were obtained from the national urban air quality real-time release platform of the China Environmental Monitoring Station. Daily data were obtained for the period from January 1, 2016, to December 31, 2021, with a total of 2192 observations. For each city, data from 2016 to 2020 were used as the training sample; 80 and 20% of the samples were used as the training and the validation sets, respectively. The data from 2021 were used as separate test sets.

### Descriptive statistics

**Figure 4:**
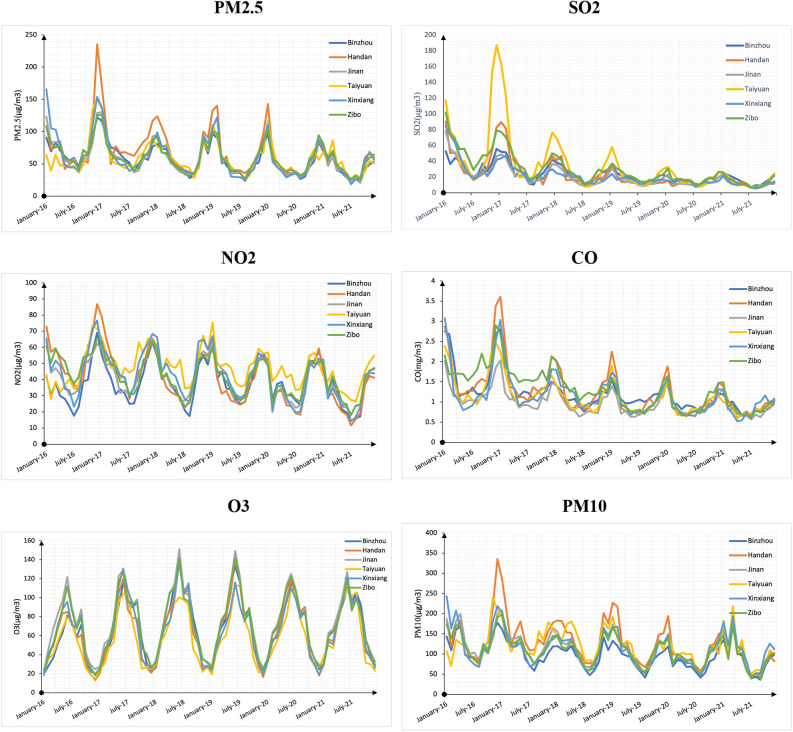
The pollutant concentrations of the six cities.

To better illustrate the situation of the used data, the pollutant concentrations of the six cities were plotted as a line graph, and the results are shown in Fig. 4. Overall, the six major air pollutants showed obvious periodicity; PM2.5, SO2, CO, and PM10 showed a yearly decreasing trend. Among them, the concentrations of PM2.5, SO2, NO2, CO, and PM10 reached their highest values in January and the lowest in September each year, showing a “U” shape; the change trend of O3 is the opposite. The highest and lowest concentrations of O3 occur in September and January every year, respectively, and the distribution is in the shape of “Λ”. This research suggests that this anomaly is not a coincidence, and a deeper connection exists between the six pollutants. In summer, strong solar radiation causes the surface temperature to rise sharply and heats the air near the surface. This leads to increased convection and precipitation, which accelerate the diffusion and deposition of atmospheric pollutants^67^. Frequent sandstorms cause air pollution^68^. Stable weather and biomass combustion are common^69^. In winter, the low surface temperature causes surface inversion, and the meteorological conditions are not conducive to vertical convection^70^; therefore, the near-surface air pollution is high. As these six pollutants have similar influencing factors and trends, the remaining five pollutants need to be combined to predict the concentration of a single pollutant.

## Results and discussion

Through knowledge of past forecasting studies^71–75^, we know that the prediction work based on LSTM obeys a particular framework: PM2.5 (or other time series) is decomposed into several IMFs and a residual by EMD; subsequently, the LSTM model is applied to each IMF and residual; and finally, the training results are simply added to obtain the predicted value. However, this framework has some limitations:

1. The inability to prevent the transfer of white noise from high frequency to low frequency during EMD decomposition.
2. Choosing the high-frequency IMF.
3. Unable to choose the optimal parameter combination in the LSTM model.
4. Decomposition predictions only for a single sequence without considering whether other factors will influence the prediction results.

To address these problems, we combine the model in this section to provide the results and discussion.

### CEEMDAN decomposition results of PM2.5

**Figure 5:**
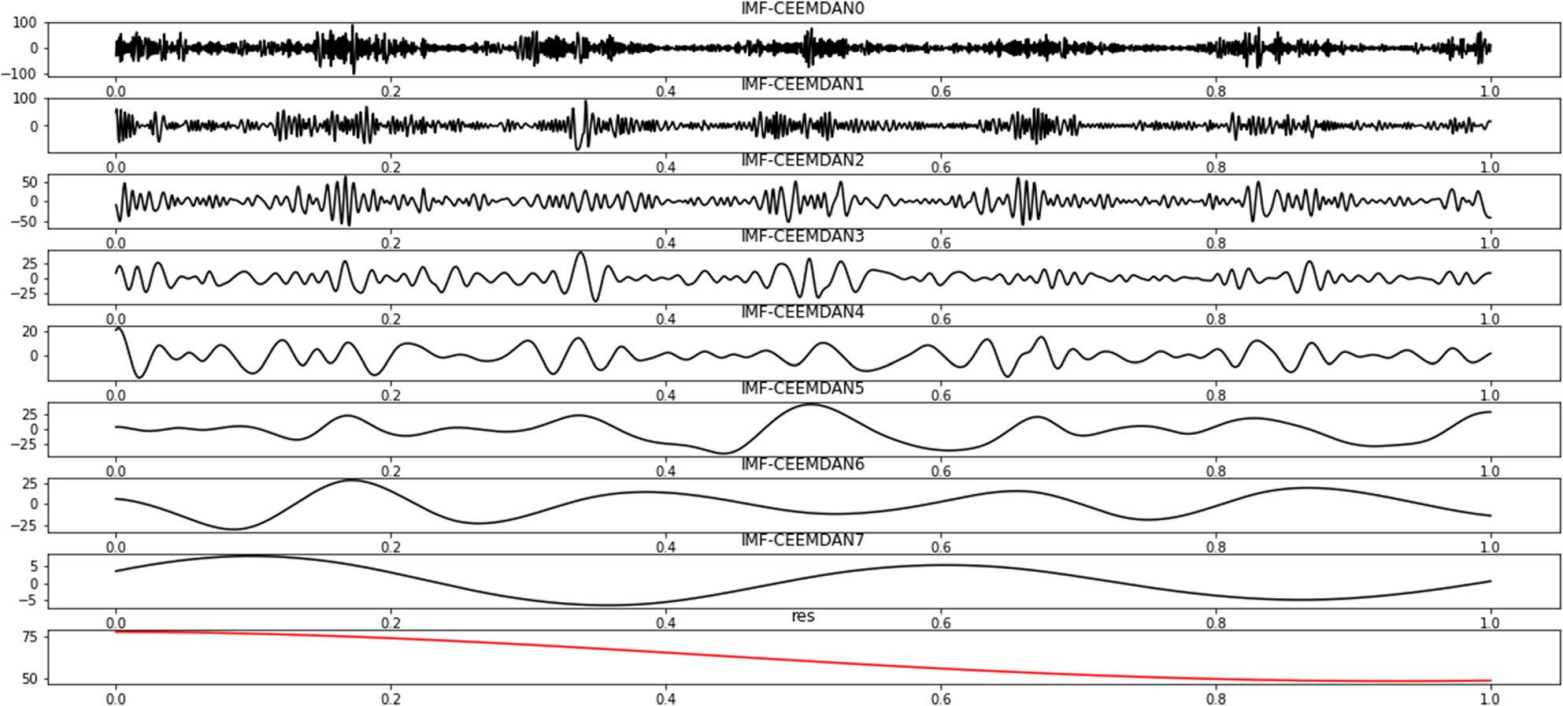
Binzhou's PM2.5 decomposition results.

The CEEMDAN algorithm was selected to solve the problem occurring when the white noise of the EMD algorithm transfers from high frequency to low frequency. Figure 5 shows the CEEMDAN decomposition results of PM2.5 of Binzhou from January 1, 2016, to December 31, 2021 (see appendix for the results of the remaining five cities). The IMF decomposed by CEEMDAN shows a certain change law and cycle and subsequently reflects the information on different time scales in the original time series. In the figure, the abscissa represents the time sequence number and the ordinate represents the frequency of each IMF and RES. The results show that in the high-frequency space (IMF0-IMF3), the IMF component fluctuates significantly and the fluctuation rate is slow, indicating that the short-term PM2.5 concentration is extremely unstable. In the intermediate frequency space (IMF4, IMF5), the IMF component exhibits a certain periodicity, and the component fluctuation frequency gradually decreases. In the low-frequency space (IMF6, IMF7, RES), the fluctuation of the IMF component becomes gentler, indicating that since 2016, PM2.5 in Binzhou has continued to decline, and the air quality has significantly improved. However, with the passage of time, the PM2.5 has decreased. The rate slowed and the concentration data smoothed out. Finally, the prediction results of the model under CEEMDAN, EEMD, VMD and EMD decompositions are presented in Table 2. Among them, the RMSE and MAE under CEEMDAN decomposition are 55.20 and 44.54% lower than EMD, respectively, and the R^2^ increased by 19.62%. In general, compared with the EMD, EEMD and VMD, CEEMDAN has a more obvious effect on improving the model prediction accuracy.

**Table 2:** Comparison of prediction accuracy between different decompositions.

Model	RMSE	MAE	R^2^
CEEMDAN-PSO-CNNLSTM	12.67541	9.60255	0.87771
EMD-PSO-CNNLSTM	19.67235	13.87995	0.70546
EEMD-PSO-CNNLSTM	19.76978	14.28564	0.70695
VMD-PSO-CNNLSTM	16.26797	10.88799	0.78680

### PSO parameter optimization results

In this study, the parameter space was chosen as the time step (n) of the time series and the number of neurons (cells) in the LSTM neural network model. The range of n was 1–20, and the range of cells was 1–100. We considered the daily data of 2016–2020 and 2021 as the training and test sets, respectively, and the parameter results of the PM2.5 prediction model training for the six cities are presented in Table 3. As presented in Table 3, among the six cities, the optimal time step for the six cities is only two and four, which shows that the CEEMDAN-PSO-CNNLSTM model constructed in this study is only dependent on data from the past few days. Additionally, the minimum and maximum numbers of LSTM neurons were 42 and 92, respectively, indicating that the model was more sensitive to changes in the number of parameter neurons.

**Table 3:** Parameter training results.

City	Cells	n
Binzhou	92	2
Jinan	51	4
Handan	42	2
Taiyuan	93	4
Xinxiang	66	2
Zibo	90	4

### Final model predictions

After CEEMDAN decomposition and PSO algorithm optimization, the variables and parameters were input into the CNN-LSTM model to predict the final air pollutant value. In other studies, prediction methods combined with EMD treated the first high-frequency IMF sequence as a noise term and discarded it, which did not contribute to the prediction result^76,77^. This method is simple and crude and may lose some useful information and retain some noise signals. Therefore, a CNN was selected to screen the IMFs. Simultaneously, to illustrate the robustness and superiority of the proposed model, the air pollution results of six types of cities affected by different air pollutants were predicted, respectively, and those of SVM, CEEMDAN-SVM, PSO-LSTM, PSO-CNN-LSTM, CEEMDAN- PSO-LSTM, CEEMDAN-PSO-CNNLSTM models were compared with each other. For conciseness, the following subsections only provide the prediction and comparison results of PM2.5, and appendix presents the prediction results of the other five pollutants.

**Figure 6:**
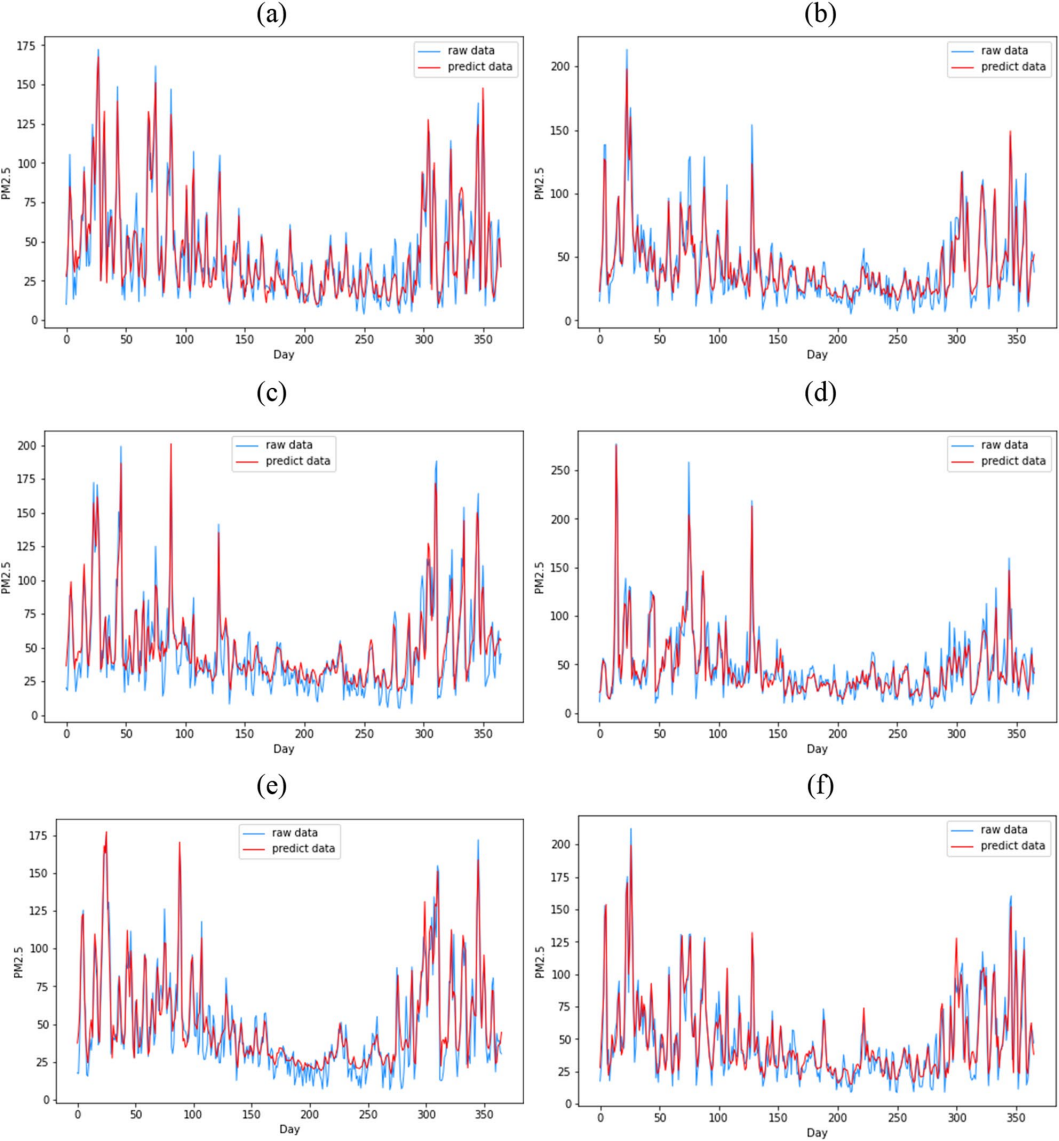
PM2.5 forecast curve for the six cities. (μg/m^3^). (**a**) Binzhou, (**b**) Jinan, (**c**) Handan, (**d**) Taiyuan, (**e**) Xinxiang and (**f**) Zibo.

The MSE, MAE, and R^2^ values obtained using the six prediction models are listed in Table 4. Overall, the proposed CEEMDAN-PSO-CNNLSTM model has the best prediction accuracy; it has the smallest MSE and MAE and the highest R2 among the predictions for the six cities. Simultaneously, Fig. 6 also shows that the PM2.5 prediction curve of the proposed model has a high degree of fit with the actual curve, and the prediction accuracy is high. Therefore, the proposed model is considered to be effective and robust in predicting results under different polluted environments and outperforms the other models.

**Table 4:** The statistical evaluation of different model performances (PM2.5).

City	Model	RMSE	MAE	R^2^
Binzhou	SVM	21.25	19.86	0.66
	CEEMDAN-SVM	18.65	17.11	0.73
	BP	19.36	12.65	0.61
	MLP	22.95	15.56	0.58
	PSO-LSTM	26.94	18.97	0.45
	PSO-CNN-LSTM	26.32	18.68	0.47
	CEEMDAN-PSO-LSTM	17.69	12.89	0.76
	CEEMDAN-PSO-CNNLSTM	12.68	9.60	0.86
Jinan	SVM	23.13	21.34	0.52
	CEEMDAN-SVM	21.52	19.90	0.59
	BP	19.90	13.03	0.58
	MLP	30.00	20.84	0.46
	PSO-LSTM	24.28	16.48	0.47
	PSO-CNN-LSTM	23.94	16.47	0.49
	CEEMDAN-PSO-LSTM	16.04	11.21	0.77
	CEEMDAN-PSO-CNNLSTM	11.01	8.41	0.87
Handan	SVM	23.52	21.17	0.67
	CEEMDAN-SVM	16.14	13.20	0.86
	BP	26.57	17.69	0.39
	MLP	27.14	18.93	0.38
	PSO-LSTM	31.48	21.49	0.46
	PSO-CNN-LSTM	31.16	21.72	0.47
	CEEMDAN-PSO-LSTM	22.31	15.10	0.72
	CEEMDAN-PSO-CNNLSTM	12.94	9.99	0.88
Taiyuan	SVM	23.55	21.91	0.62
	CEEMDAN-SVM	19.86	17.92	0.73
	BP	30.55	16.26	0.25
	MLP	27.47	19.24	0.37
	PSO-LSTM	30.79	20.63	0.44
	PSO-CNN-LSTM	27.31	18.81	0.48
	CEEMDAN-PSO-LSTM	20.79	15.16	0.70
	CEEMDAN-PSO-CNNLSTM	12.38	9.33	0.88
Xinxiang	SVM	23.68	21.43	0.53
	CEEMDAN-SVM	18.53	14.85	0.71
	BP	725.29	19.29	0.34
	MLP	28.44	20.65	0.36
	PSO-LSTM	23.91	17.52	0.52
	PSO-CNN-LSTM	23.29	18.09	0.55
	CEEMDAN-PSO-LSTM	16.00	11.53	0.79
	CEEMDAN-PSO-CNNLSTM	11.63	8.98	0.88
Zibo	SVM	19.95	18.36	0.70
	CEEMDAN-SVM	19.70	17.84	0.71
	BP	23.49	16.96	0.48
	MLP	24.57	18.16	0.43
	PSO-LSTM	26.89	18.26	0.46
	PSO-CNN-LSTM	24.61	17.00	0.55
	CEEMDAN-PSO-LSTM	19.77	14.29	0.71
	CEEMDAN-PSO-CNNLSTM	10.66	8.34	0.89

Specifically, the prediction accuracy of the model after CEEMDAN decomposition was significantly higher than those of the models without decomposition (CEEMDAN-PSO-CNNLSTM vs. PSO-CNN-LSTM, CEEMDAN-PSO-LSTM vs. PSO-LSTM, and CEEMDAN-SVM vs. SVM). Considering Binzhou as an example, the prediction accuracies of the SVM, PSO-LSTM, and PSO-CNNLSTM models decomposed by CEEMDAN improved by 0.08, 0.31, and 0.39 (R^2^), respectively, while the prediction errors were reduced by 12.24, 34.34, and 51.82%, (RMSE) and 13.85, 32.05, and 48.61% (MAE), respectively. This shows that the signal decomposition technique can effectively reduce the non-stationarity of the PM2.5, thereby improving the performance. Additionally, according to the results listed in Table 2, the necessity of CEEMDAN decomposition is also confirmed. Finally, in response to the second question raised at the beginning of this section, the model prediction results are compared and analyzed. We found that when CEEMDAN is not decomposed or the model has few input variables (only five variables are input in the PSO-LSTM and PSO-CNN-LSTM models), the improvement in prediction accuracy by using CNN for feature screening is not obvious. In the six selected cities, the R^2^ values increased by approximately 0.01–0.08, and the RMSE and MAE decreased by approximately 1–8% and 1–9%, respectively. For the model after CEEMDAN decomposition (there are more than 10 variables in the CEEMDAN-PSO-LSTM and CEEMDAN-PSO-CNNLSTM models), the use of CNN for feature screening greatly improved the model prediction accuracy; in the six selected cities, the R^2^ increased by 0.10–0.18, and the RMSE and MAE decreased by approximately 27–46% and 22–42%, respectively. We concluded that when there are more variable inputs, using a CNN for feature screening can increase the accuracy and error of the model. Therefore, it is feasible and effective to use a CNN to screen each IMF component after CEEMDAN decomposition.

**Figure 7:**
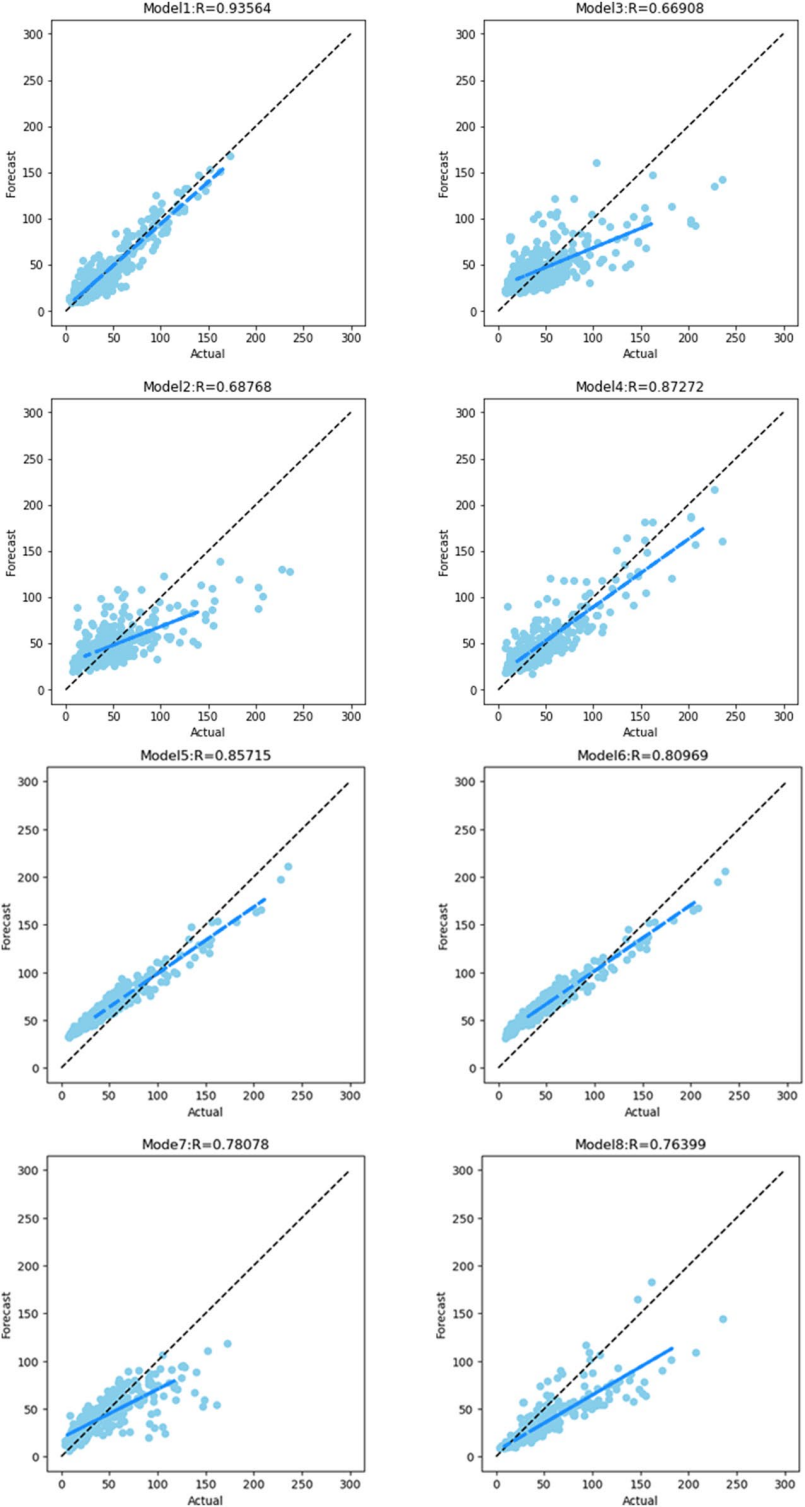
Scatterplots of the actual and forecast PM2.5 values achieved using various models (Binzhou data). Model 1: CEEMDAN-PSO-CNNLSTM. Model 2: PSO-LSTM. Model 3: PSO-CNN-LSTM. Model 4: CEEMAND-PSO-LSTM. Model 5: CEEMDAN-SVM. Model 6: SVM. Model 7: BP. Model 8: MLP.

The PM2.5 prediction scatter plot for each model is shown in Fig. 7. The proposed model had the highest R-value (0.94). The graph shows that CEEMDAN-PSO-CNNLSTM also shows a fitting advantage over the other models. The scattered points are evenly distributed on both sides of the diagonal, and the fitted straight line is the closest to the diagonal. Additionally, although the R-value of the SVM model was high, based on the results of the model prediction, the predicted value of the SVM was usually high, and it was not sensitive to changes in extreme values. Overall, the proposed model showed better PM2.5 predictions and achieved better prediction performance.

### Comparison of prediction results without introducing air pollutants

**Figure 8:**
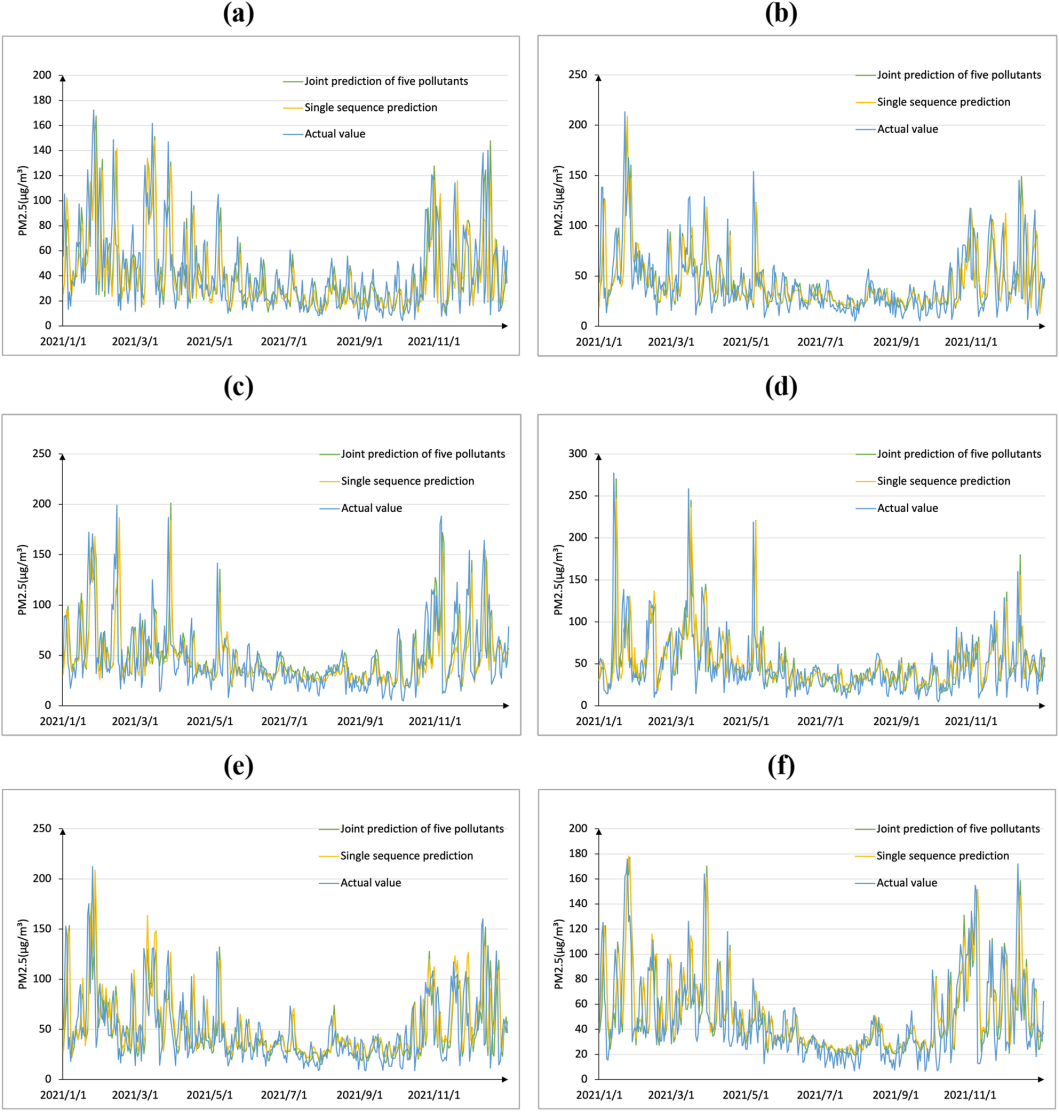
PM2.5 prediction result curves (with air pollutants vs. without air pollutants). (**a**) Binzhou, (**b**) Jinan, (**c**) Handan, (**d**) Taiyuan, (**e**) Xinxiang and (**f**) Zibo.

The prediction results for PM2.5 prediction results of the six cities are shown in Fig. 8. From the graph, the curve of the joint prediction of the five pollutants has a high degree of fit with the actual curve, which can better reflect the change trend of PM2.5 and changes in extreme values. The RMSE, MAE, and R^2^ values obtained from the two predictions are listed in Table 5. Considering the prediction of PM2.5, RMSE and MAE of Binzhou decreased by 10.70 and 7.69%, respectively, and R^2^ increased by 0.07; the RMSE and MAE of Jinan decreased by 9.98 and 11.19%, respectively, and R^2^ increased by 0.03; the RMSE and MAE of Handan decreased by 15.92 and 11.04%, respectively, and R^2^ increased by 0.09; the RMSE and MAE of Taiyuan decreased by 14.27 and 16.09%, respectively, and R^2^ increased by 0.05; the RMSE and MAE of Xinxiang decreased by 16.81 and 15.20%, respectively, and R^2^ increased by 0.07; the RMSE and MAE of Zibo decreased by 27.78 and 24.80%, respectively, and R^2^ increased by 0.1. Notably, compared with those of the single PM2.5 time-series prediction, the RMSE and MAE obtained by the combination of the other five pollutants were smaller, and the R^2^ was larger. This indicated that the input of the five pollutant data improved the prediction accuracy of the model. The positive effect, especially for the cities not polluted by PM2.5, by combining the five air pollutants to predict the model performance was more significant; however, for the cities mainly polluted by PM2.5, introducing the remaining five air pollutants into the model had a more significant impact. Various pollutants also have a certain effect on improving the prediction accuracy. Therefore, it is necessary to combine the predictions of the remaining five pollutants with that of the sixth air pollutants.

**Table 5:** Comparison of the accuracy of prediction results before and after the introduction of air pollutants.

City	Joint forecast(with air pollutants)			Single sequence prediction(without air pollutants)		
	RMSE	MAE	R^2^	RMSE	MAE	R^2^
Binzhou	12.68	9.60	0.86	14.20	10.40	0.79
Jinan	11.01	8.41	0.87	12.23	9.47	0.84
Handan	12.94	9.99	0.88	15.39	11.23	0.79
Taiyuan	12.38	9.33	0.88	14.44	11.12	0.83
Xinxiang	11.63	8.98	0.88	13.98	10.59	0.81
Zibo	10.66	8.34	0.89	14.76	11.09	0.79

### Health effect assessment

Air pollution affects the economy and causes serious damage to human health. The number of deaths due to excessive pollutant concentrations is presented in Table 8. This section is based on the predicted concentrations in 2021, combined with historical research, and uses the WHO revised guidance value in 2021, the first-level limit, and the second-level limit of China's “Environmental Quality Standard” as the reference concentrations to evaluate the health impact of the population. Tables 6 and 7 the aggregated air pollutant concentration reference values and percentage increases in the population mortality, respectively. Notably, the air pollutants that significantly increase the mortality rate of the population are NO2, which increases the mortality rate by 1.4% per 10 μg·m^−3^, followed by SO2, which increases the mortality rate by 0.9% per 10 μg·m^−3^. As presented in Table 8, under the latest WHO standard, the number of deaths due to excessive NO2 concentration is the largest among the six cities, followed by those due to PM10 and PM2.5, respectively. According to the national standard, the number of deaths due to excessive PM10 is the largest, followed by PM2.5. In general, although the concentrations of SO2, CO, and O3 in some cities are significantly higher than those in others, PM2.5, PM10 are the main air pollutants affecting human health. PM2.5, PM10, and NO2 should be considered as the focus of air pollution prevention and control, and must be simultaneously combined with the city's own SO2, CO, and O3 concentration characteristics to gradually tighten the standard limit.

**Table 6:** Reference levels of various air pollutants.

	PM2.5 (μg·m^−3^)	PM10 (μg·m^−3^)	O3 (μg·m^−3^)	NO2 (μg·m^−3^)	SO2 (μg·m^−3^)	CO (mg·m^−3^)
WHO guideline value	5	15	100	10	40	4
National first-class standard	15	40	100	40	20	4
National second-class standard	35	70	160	40	60	4

**Table 7:** Percentage increase in the population mortality due to excessive pollutant concentrations.

Air pollutants	ER (%)	95%CI (%)	Source
PM2.5 (10 μg·m^−3^)	0.38	0.31 ~ 0.45	^78^
PM10 (10 μg·m^−3^)	0.31	0.22 ~ 0.41	^79^
O3 (10 μg·m^−3^)	0.40	0.30 ~ 0.50	^80^
NO2 (10 μg·m^−3^)	1.40	1.10 ~ 1.60	^81^
SO2 (10 μg·m^−3^)	0.90	0.60 ~ 1.10	^81^
CO (1 mg·m^−3^)	3.70	2.88 ~ 4.51	^78^

**Table 8:** Number of deaths that could be avoided by meeting air pollution standards in 2021 (10,000 people).

Air pollutants	Binzhou	Jinan	Handan	Taiyuan	Xinxiang	Zibo
**WHO guideline value**						
PM2.5	4.81 (95% CI: 3.93 ~ 5.70)	16.47 (95% CI: 13.44 ~ 19.51)	15.59 (95% CI: 12.72 ~ 18.46)	7.48 (95% CI: 6.10 ~ 8.85)	9.57 (95% CI: 7.81 ~ 11.33)	7.81 (95% CI: 6.37 ~ 9.25)
PM10	6.85 (95% CI: 4.86 ~ 9.07)	22.13 (95% CI: 15.70 ~ 27.26)	23.49 (95% CI: 16.67 ~ 31.07)	12.75 (95% CI:9.05 ~ 16.86)	16.64 (95% CI: 11.81 ~ 22.00)	10.80 (95% CI: 7.67 ~ 14.29)
O3	0.00	0.00	0.00	0.00	0.00	0.00
NO2	9.18 (95% CI: 3.93 ~ 10.49)	28.44 (95% CI: 12.19 ~ 32.50)	24.09 (95% CI: 10.32 ~ 27.53)	18.20 (95% CI: 7.80 ~ 20.80)	17.41 (95% CI: 7.46 ~ 19.90)	16.09 (95% CI: 6.90 ~ 18.39)
SO2	0.00	0.00	0.00	0.00	0.00	0.00
CO	0.00	0.00	0.00	0.00	0.00	0.00
**National first-class standard**						
PM2.5	3.53 (95% CI: 2.88 ~ 4.18)	13.09 (95% CI: 10.68 ~ 15.50)	11.97 (95% CI: 9.77 ~ 14.18)	5.75 (95% CI: 4.69 ~ 6.81)	7.36 (95% CI: 6.00 ~ 8.72)	6.03 (95% CI: 4.92 ~ 7.14)
PM10	4.23 (95% CI: 3.00 ~ 5.89)	15.22 (95% CI: 10.80 ~ 20.13)	16.11 (95% CI: 11.43 ~ 21.31)	9.23 (95% CI: 6.55 ~ 12.21)	12.13 (95% CI: 8.61 ~ 16.05)	7.16 (95% CI: 5.08 ~ 9.47)
O3	0.00	0.00	0.00	0.00	0.00	0.00
NO2	0.00	0.00	0.00	0.00	0.00	0.00
SO2	0.00	0.00	0.00	0.00	0.00	0.00
CO	0.00	0.00	0.00	0.00	0.00	0.00
**National secondary standard**						
PM2.5	0.95 (95% CI: 0.77 ~ 1.12)	6.32 (95% CI: 5.15 ~ 7.49)	4.73 (95% CI: 3.86 ~ 5.6)	2.30 (95% CI: 1.87 ~ 2.72)	2.95 (95% CI: 2.40 ~ 3.49)	2.46 (95% CI: 2.00 ~ 2.91)
PM10	1.07 (95% CI: 0.76 ~ 1.42)	6.94 (95% CI: 4.92 ~ 9.18)	7.25 (95% CI: 5.15 ~ 9.59)	5.00 (95% CI: 3.55 ~ 6.62)	6.73 (95% CI: 4.78 ~ 8.90)	2.80 (95% CI: 1.98 ~ 3.70)
O3	0.00	0.00	0.00	0.00	0.00	0.00
NO2	0.00	0.00	0.00	0.00	0.00	0.00
SO2	0.00	0.00	0.00	0.00	0.00	0.00
CO	0.00	0.00	0.00	0.00	0.00	0.00

## Conclusions

Cities are usually affected by air pollution in several ways. To promote the sustainable development of urban public health and the sustainable development of society, a stable and high-precision air pollutant prediction model is required. This paper studied a series of existing LSTM prediction frameworks and found that some problems still exist in the existing prediction frameworks, including the selection of high-frequency feature signals, the selection of LSTM model parameters, and predictions without considering other closely related drivers of air pollution. Therefore, this study develops a hybrid model named CEEMDAN-PSO-CNNLSTM to solve these problems. First, CEEMDAN is used to decompose the air pollutant signal, and the decomposed data are then sent to the CNN-LSTM neural network for PSO optimization. Finally, the optimized parameter input model was trained using the original data to obtain the final prediction result. Combined with the evaluation criteria, the proposed model had the highest accuracy among the six compared models. Additionally, predictions were made for six cities affected by different pollutants, and we found that the prediction accuracy of the proposed model was the highest in each comparison, indicating the robustness of the model. The advantages of the proposed hybrid model are as follows: 1. considering the influence of other air pollutants, the prediction accuracy for a single air pollutant was improved. 2. Combining the CEEMDAN decomposition with the PSO algorithm and using CNN to screen the IMF not only solves the problem of parameter selection in the LSTM model, but also solves that of white noise and high-frequency signals interfering with the prediction results; thus, it realized the improvement of the traditional prediction framework. At the end of the article, we predict the degree of harm that air pollutants may bring to the health of the population, and offer some suggestions. However, the model proposed in this study still has room for optimization. For example, we consider the spatial location information of each forecast station and improve the prediction accuracy through the joint prediction of different sites. Additionally, referring to forecasting work in other areas, there are still many variables that have not been added to the process of predicting pollutants, such as the wind speed, air pressure, humidity, and temperature, are also important factors affecting the air quality^82,83^. In future studies, the prediction of air pollution will be further refined, and other variables that may affect air pollution will be added to further optimize the hybrid model and improve its effectiveness.

## Supplementary Information

Supplementary Information 1.

Supplementary Information 2.

Supplementary Information 3.

Supplementary Information 4.

Supplementary Information 5.

Supplementary Information 6.

## Data Availability

All data generated or analyzed during this study are included in this its supplementary information files.
