# Supplementary material for: A novel hybrid model for six main pollutant concentrations forecasting based on improved LSTM neural networks
Source: Sci Rep. 2022 Aug 24;12:14434. doi: 10.1038/s41598-022-17754-3 (PMC9402967; doi:10.1038/s41598-022-17754-3)
Supplement: Supplementary file 1 — Supplementary Information 1. [file 41598_2022_17754_MOESM1_ESM.docx]

**Appendix**


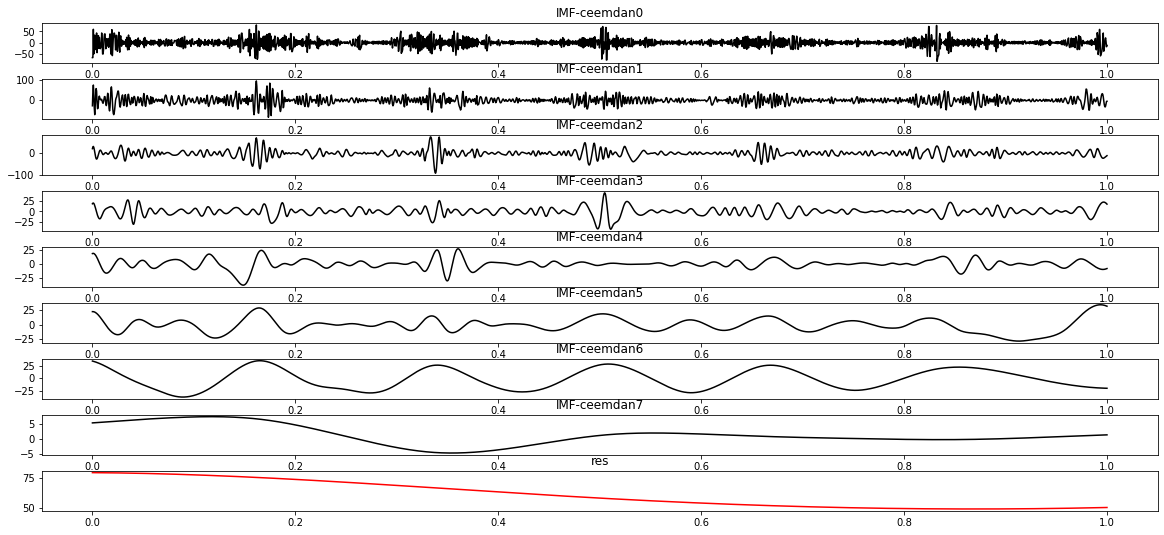


Fig.1 Jinan's PM2.5 decomposition results.


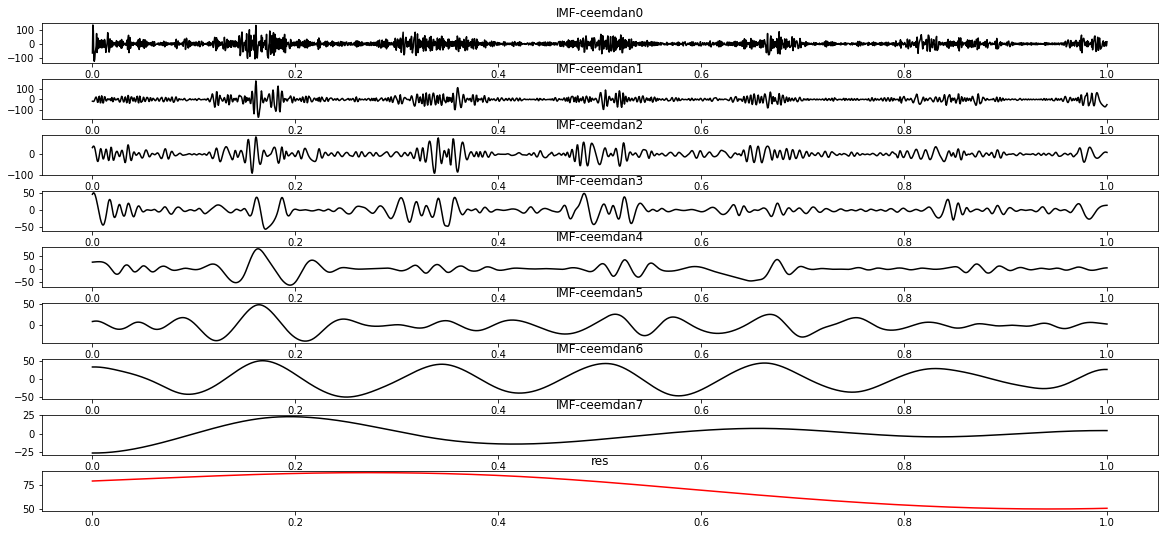


Fig.2 Handan's PM2.5 decomposition results.


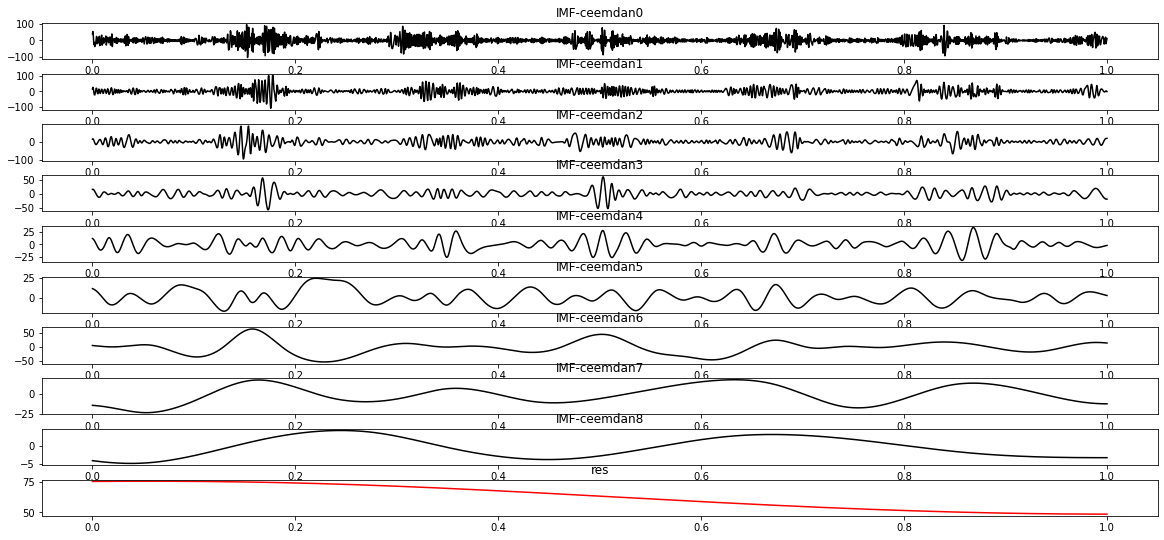


Fig.3 Taiyuan's PM2.5 decomposition results.


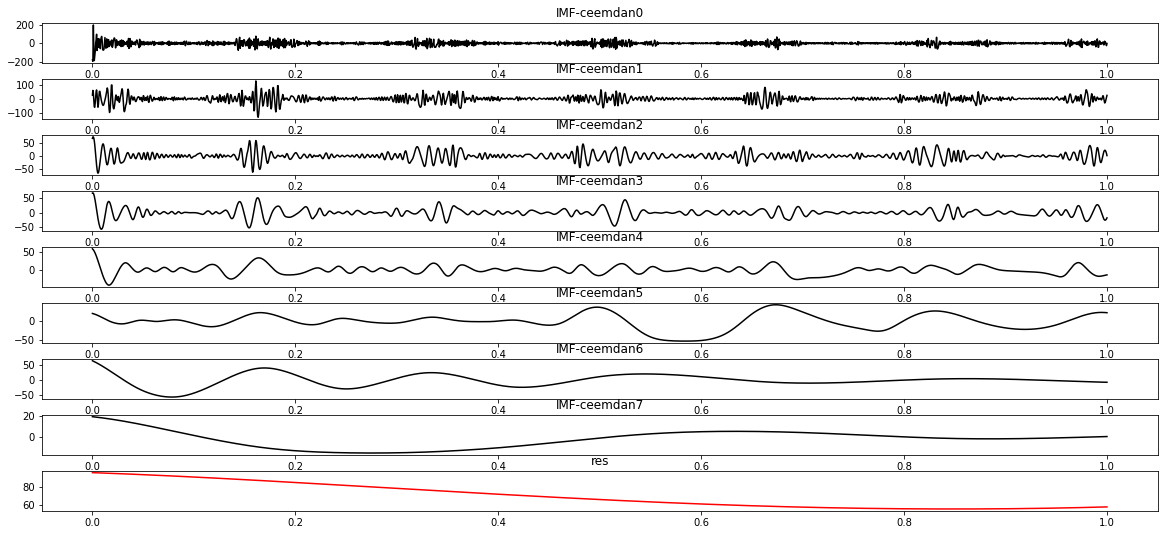


Fig.4 Xinxiang's PM2.5 decomposition results.


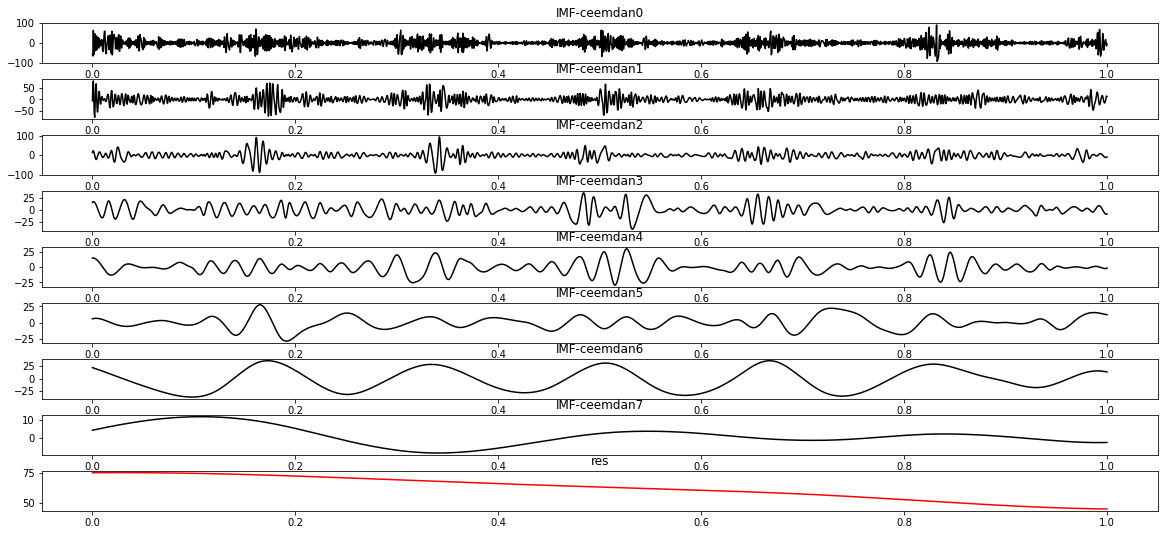


Fig.5 Zibo's PM2.5 decomposition results.

| (a) | (b) |
| --- | --- |
| 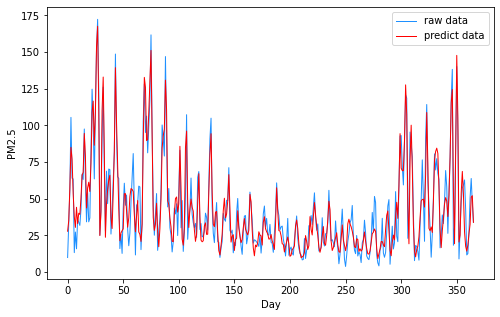 | 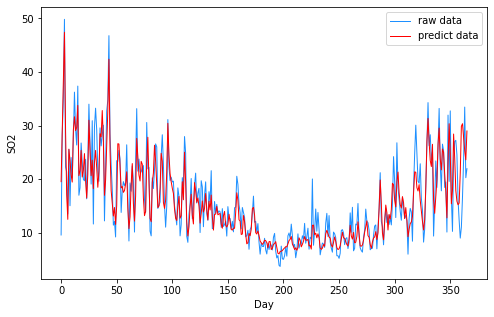 |
| (c) | (d) |
| 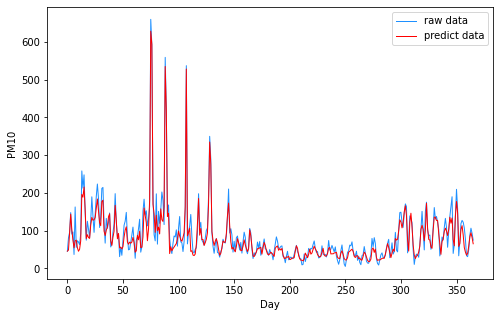 | 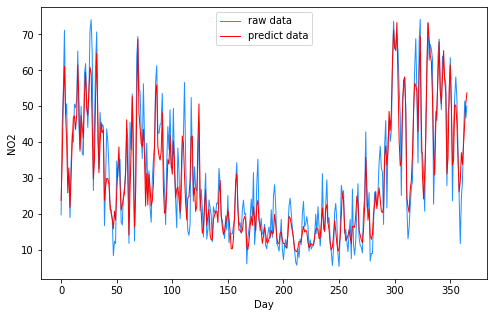 |
| (e) | (f) |
| 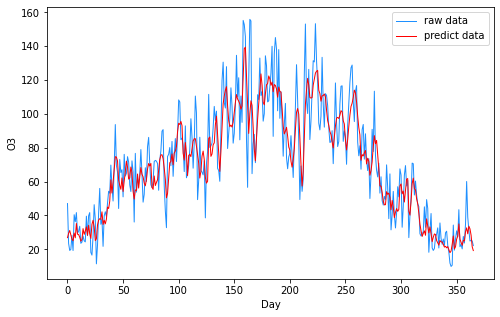 | 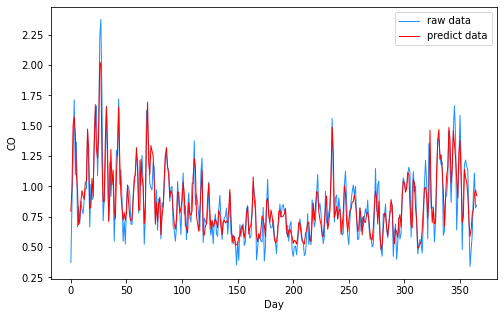 |

1. PM2.5, (b) SO2, (c) PM10, (d) NO2, (e) O3, and (f) CO.

Fig.6 Prediction curves for six air pollutants in Binzhou.
